# *hetN* and *patS* Mutations Enhance Accumulation of Fatty Alcohols in the *hglT* Mutants of *Anabaena* sp. PCC 7120

**DOI:** 10.3389/fpls.2020.00804

**Published:** 2020-07-08

**Authors:** Heli Siti Halimatul Munawaroh, Egi Tritya Apdila, Koichiro Awai

**Affiliations:** ^1^Laboratory of Chemistry Study Program, Department of Chemistry Education, Universitas Pendidikan Indonesia, Bandung, Indonesia; ^2^Graduate School of Science and Technology, Shizuoka University, Shizuoka, Japan; ^3^Research Institute of Electronics, Shizuoka University, Hamamatsu, Japan; ^4^Department of Biological Science, Faculty of Science, Shizuoka University, Shizuoka, Japan

**Keywords:** *Anabaena* sp. PCC 7120, heterocyst, heterocyst-specific glycolipids, fatty alcohol, aeration

## Abstract

The heterocysts present in filamentous cyanobacteria such as *Anabaena* sp. PCC 7120 are known to be regulated by HetN and PatS, the repressors of heterocyst differentiation; therefore, the inactivation of these proteins will result in the formation of multiple heterocysts. To enhance the accumulation of fatty alcohols synthesized in the heterocyst, we introduced mutations of these repressors to increase heterocyst frequency. First, we isolated double mutants of *hetN* and *patS* and confirmed that the null mutation of these genes promoted higher frequencies of heterocyst formation and higher accumulation of heterocyst-specific glycolipids (Hgls) compared with its wild type. Next, we combined *hetN* and *patS* mutations with an *hglT* (encoding glycosyltransferase, an enzyme involved in Hgl synthesis) mutation to increase the accumulation of fatty alcohols since knockout mutation of *hglT* results in accumulation of very long chain fatty alcohol, the precursor of Hgl. We also observed retarded growth, lower chlorophyll content and up to a five-fold decrease in photosynthetic activity of the *hetN*/*patS*/*hglT* triple mutants. In contrast, the triple mutants showed three times higher heterocyst formation frequencies than the *hglT* single mutant and wild type. The production rate of fatty alcohol in the triple mutants attained a value 1.41 nmol/mL OD_730_, whereas accumulation of Hgls in the wild type was 0.90 nmol/mL OD_730_. Aeration of culture improved the accumulation of fatty alcohols in *hetN*/*patS*/*hglT* mutant cells up to 2.97 nmol/mL OD_730_ compared with cells cultured by rotation. Our study outlines an alternative strategy for fatty alcohol production supported by photosynthesis and nitrogen fixation.

## Introduction

*Anabaena* sp. PCC 7120 (hereinafter Anabaena) is a multicellular cyanobacterium that possesses a long filament comprising 100 or more vegetative cells usually in the presence of combined nitrogen sources in the medium. When the combined nitrogen concentration in the environment reduces, Anabaena develops heterocyst cells to separate oxygen-labile nitrogen fixation from oxygen produced due to photosynthesis in the vegetative cells. Heterocyst cells are typically distinguishable from vegetative cells by their morphology, such as a large and round shape, reduction of pigments, thick cellular envelopes, and cyanophycin granules at poles adjacent to the vegetative cells. These cells are surrounded by a glycolipid layer to provide a micro-oxic environment that protects the enzyme nitrogenase from oxygen diffusion from outside the cells (Ernst et al., 1992; Fay, 1992; Wolk, 1996; Maldener et al., 2003; Huang et al., 2004; Awai et al., 2009).

Numerous proteins have been found to be involved in heterocyst development, including repressors. In Anabaena, HetR is a well-known protein that is involved in the early stage of heterocyst formation. PatS and HetN are known to play a prominent role in heterocyst development and its formation pattern maintenance (Buikema and Haselkorn, 1991; Golden and Yoon, 2003). Both *patS* and *hetN* have been reported to encode a diffusible inhibitor of differentiation, the RGSGR pentapeptide, which affects heterocyst formation pattern. The product of *patS* is proposed to control heterocyst formation through *hetR* regulation. The *patS* gene product is thought to function through the means of cell-to-cell signaling to prevent the formation of multiple contiguous heterocysts. The pattern of heterocyst formation possibly requires the interaction between HetR and PatS (Huang et al., 2004). PatS diffuses laterally to inhibit the differentiation of neighboring cells by hampering the DNA binding activity of HetR (Golden and Yoon, 2003; Zhang et al., 2006). Another gene, *hetN*, is required for the maintenance of the heterocyst pattern (Callahan and Buikema, 2001). Unlike *patS* mutants, the deletion of *hetN* in Anabaena shows no alteration in the heterocyst formation pattern and the vegetative cell interval. However, this null mutant also forms multiple heterocysts after the normal pattern of heterocyst formation (Fan et al., 2005), indicating that HetN is not required for the formation of the initial heterocyst pattern in response to nitrogen-starved conditions. Yet, this gene plays an important role in the maintenance of the heterocyst pattern (Callahan and Buikema, 2001).

Borthakur et al. (2005) analyzed the effects of *patS* and *hetN* mutations on heterocyst development. They found that the double mutant of *patS* and *hetN* had significantly higher heterocyst frequency than the single mutant of *patS* or *hetN*, suggesting that *patS* and *hetN* suppress heterocyst differentiation by different pathways. Surprisingly, the inactivation of both genes leads to the differentiation of almost all vegetative cells into heterocyst cells under nitrogen-starved conditions (Borthakur et al., 2005). This double mutant was not a null mutant; the inducible repression system for *hetN* was used for this procedure because it was thought that the double mutant is lethal under nitrogen-starved conditions. Afterward, Corrales-Guerrero et al. (2014) reported the double null mutant of *patS* and *hetN* in Anabaena. However, a recent study found that substrains of Anabaena maintained separately in different laboratories have higher number of genomic variations, such as SNPs, and these polymorphisms change the heterocyst differentiation patterns (Wang et al., 2018).

As described above, to prevent oxygen diffusion into the heterocyst, their cellular envelope develops a structural barrier called the heterocyst-specific glycolipid layer (HGL). HGL is composed of heterocyst-specific glycolipids (Hgls) that comprise glucose attached to a very long chain fatty alcohol by an ether bond. The final step of Hgl synthesis is catalyzed by the enzyme glucosyltransferase HglT (Awai and Wolk, 2007). The null mutant of *hglT* differentiates into heterocysts but fails to form the HGL and instead accumulates fatty alcohol (Halimatul et al., 2014). The accumulation of fatty alcohols in the *hglT* mutants could be of biotechnological interest that may help in finding an alternative route for fatty alcohol production. Long chain hydrocarbons including long chain fatty alcohols are nowadays drawing more attention due to their high energy density, low moisture absorption, low vitality, and compatibility with existing engines and transport facilities. However, fatty alcohol is mostly prepared from natural oil through the processes of transesterification and hydrogenation. These processes require harsh production environments or introduce harmful materials into the environment (Zheng et al., 2012). For these reasons, researchers are on the lookout for organisms that efficiently produce fatty alcohols. Recently, the production of fatty acid ethyl esters and fatty alcohols have been reported in genetically modified *Escherichia coli* (Steen et al., 2010). However, this system requires the addition of a stable carbon source which in turn increases production costs.

To enhance the accumulation of fatty alcohols, we combined the genes of mutants’ *hetN* and *patS* with that of *hglT* gene. The knockout mutants of *hetN* and *patS* formed approximately three times more heterocysts than the wild type under nitrogen-starved conditions. In the triple mutants of *hetN*, *patS*, and *hglT* genes, more fatty alcohols were accumulated compared with the single *hglT* mutant. In this study, we demonstrated that increased ratio of heterocyst per filament in the *hglT* mutants of Anabaena enhances the accumulation of fatty alcohols.

## Materials and Methods

### Growth Conditions of the Wild Type and Mutants of Anabaena

Anabaena and *hglT* mutant strains were grown in the liquid medium of BG11 (containing nitrate as a nitrogen source) (Stanier et al., 1971) at a temperature of 30°C in the presence of light (50–80 μmol m^–2^ s^–1^) on a rotary shaker (120 rpm) as described previously (Awai et al., 2007). For the nitrogen-starved condition experiments, cells were first grown in the BG11 medium to an optical density of 0.8–1.2 at 730 nm (OD_730_), washed thrice with nitrogen-free medium (BG11_0_: BG11 without nitrate), and resuspended in BG11_0_. For cultures exposed to air bubbling, 50 ml of BG11_0_ was used in a 93 ml test tube.

### Isolation of Mutants of Anabaena

The knockout vector of the *hetN* gene was constructed: DNA fragments upstream and downstream of *hetN* gene were amplified by the polymerase chain reaction (PCR) technique using the primer pairs #1 and #2 and #3 and #4, respectively (see Supplementary Table S1). The upstream fragment was cloned into the *Sma*I site of pMobΩ1 (Saito and Awai, 2020) using In-Fusion HD Cloning Kit with a Cloning Enhancer (Takara Bio, Shiga, Japan), and the downstream fragment was cloned into the *Apa*I site to construct the knockout vector, pMO1hetNKO.

The knockout vector of *patS* was also constructed by amplifying the DNA fragments upstream and downstream of *PatS*. The upstream fragment was amplified by PCR using primers #5 and #6, and the downstream fragment of *patS* was amplified using primers #7 and #8. The upstream fragment was cloned into the *Apa*I site of pMobEm1 (Awai et al., 2014) using the In-Fusion HD Cloning Kit with Cloning Enhancer, and the downstream fragment was cloned into the *Sma*I site to construct the knockout vector, pME1patSKO.

The knockout vector of the *hglT* gene was constructed as described previously (Halimatul et al., 2014). The constructed plasmid vectors were introduced consecutively into the wild type and the mutant strains of Anabaena by the triparental mating method of Elhai and Wolk (1988). The constructed vectors above contain the *sacB* gene, and we used 6% sucrose for positive selections of mutants with double recombination.

The genomic DNA from the wild type and transformed cells were used as templates for PCR genotyping with the primers described below using HybriPol DNA polymerase (Bioline). Genomic DNA was extracted from the cells macerated with glass beads in TE buffer, followed by phenol/chloroform extraction and ethanol precipitation, as seen elsewhere. PCR-based confirmation of gene disruption was performed using external and internal primers to amplify the full length of *hglT, hetN, or patS* to confirm the insertion of the antibiotic resistance gene into the target gene and for the detection of deletion of the central part of *hglT, hetN*, or *patS.* Genotyping for *hglT* gene disruption was performed using primers #9 and #10 for the amplification of full-length *hglT*, #10 and #11 for insertion of the kanamycin resistance gene into *hglT*, and #9 and #12 for detection of deletion of the central part of *hglT*.

Polymerase chain reaction-based confirmation of *hetN* deletion was conducted using primers #1 and #4 for the amplification of full-length *hetN*, #4 and #13 for insertion of the spectinomycin/streptomycin resistance gene into *hetN*, and #4 and #14 for detection of deletion of the central part of *hetN*.

Genotyping for *patS* gene deletion was performed using primers #5 and #8 for the amplification of full-length *patS*, #8 and #15 for insertion of the erythromycin resistance gene into *patS*, and #5 and #16 and #5 and#17 for detection of deletions of the central part of *patS* in the Δ*hetN/patS* double and Δ*hetN/patS*/*hglT* triple mutants, respectively.

### Chlorophyll Content, Cell Spectrum, Lipid Content, and Oxygen Evolution Analysis

Cells of each strain were precipitated from 1 mL culture (OD_730_ = 0.8–1.2), resuspended in 90% methanol and measured using the method reported by Meeks and Castenholz (1971). The absorption spectra of the cells were determined by harvesting 1 mL of the cells and resuspending in fresh BG11 medium prior to measurement. The cells were subsequently scanned from 350 to 800 nm using a spectrophotometer UV-2450 (Shimadzu, Kyoto, Japan) with an integrating sphere. The lipid content was measured as described previously (Halimatul et al., 2014).

Bulk photosynthetic activities were determined by measuring the oxygen evolution rate at a temperature of 25°C with a Clark-type oxygen electrode (Hansatech instruments, Norfolk, United Kindom) using 1 mL cell suspension of the samples (approximately 5–7 μg Chl *a*/mL). The Anabaena cells were illuminated with a halogen lamp at light intensity intervals of 0 (dark conditions), 33, 58, 113, 222, and 416 μmol photons/mg Chl/h.

### RNA Extraction, Reverse Transcription, and qRT-PCR

Total RNA was isolated from whole filaments according to the manufacturer’s protocol (RNeasy, Qiagen, Hilden, Germany) and treated with DNase I (Takara Bio, Shiga, Japan). cDNA synthesis was performed using 400 ng of purified RNA with random hexamer and PrimeScript II Reverse Transcriptase (Takara Bio) according to the manufacturer’s protocol. The generated cDNA was used as a template for qRT-PCR analysis. qRT-PCR was performed with Thermal Cycler Dice Real Time System (Takara Bio) in a 20-μl reaction mixture containing 10 μl of SYBR Premix Ex Taq (Takara Bio) and 0.4 μM each of *nifH* gene-specific forward and reverse primers (#18 and #19). Relative volume ratios were normalized with the values for *rnpB*, which encodes a subunit of RNaseP, amplified with the primer set #20 and #21. The relative quantities are represented as means of duplicate experiments.

## Results

### Deletions of *hetN* and *patS* Enhanced Heterocyst Frequency and Hgl Accumulation

**FIGURE 1:**
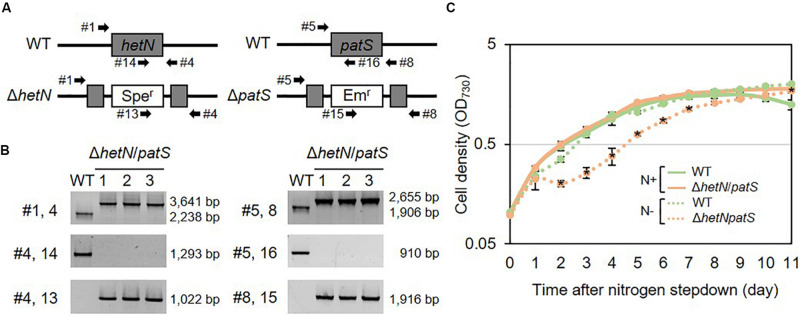
Genotype and growth curve of *hetN*/*patS* double mutants. **(A)** Schematic representation of *hetN* and *patS* regions of the wild type and mutant genomes. Spe^r^: spectinomycin, Em^r^: erythromycin resistance cassettes. **(B)** Genotype analysis of the double mutants using the primers highlighted in **(A)** by arrows. **(C)** Growth curve of the double mutants. Solid line: N+, hashed line: N–. Error bars indicate the SD based on three independent experiments. Asterisks in the markers indicate significant differences from the wild type grown under same conditions (*P* < 0.01, Welch’s *t* test).

**FIGURE 2:**
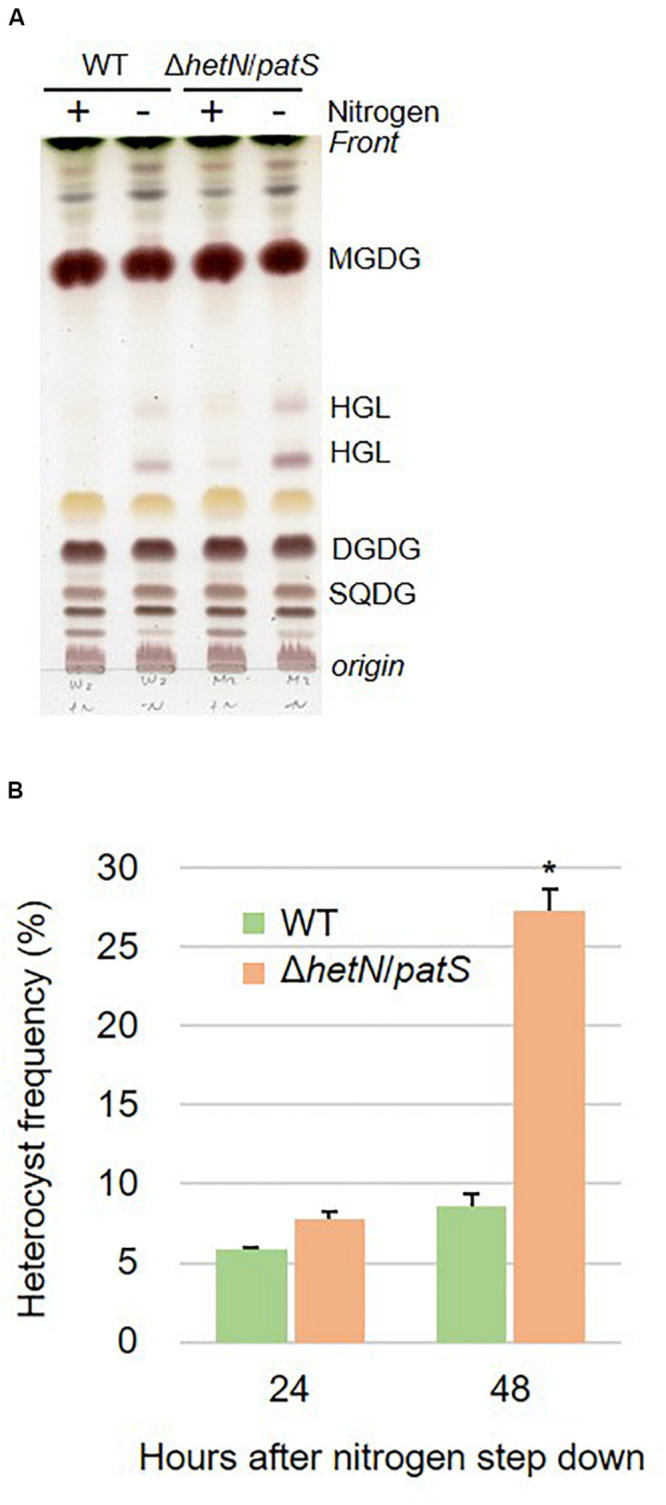
Lipid composition and heterocyst frequency of the wild type (WT) and double mutant. **(A)** Lipids separated by TLC of the wild type (WT) and the double mutant. Lipids were extracted from the cells grown in BG11 and BG11_0_ (48 h of nitrogen step-down). **(B)** Heterocyst frequency. Error bars indicate the SD based on three independent experiments. Approximately 500 cells were counted per experiment. An asterisk indicates a significant difference from the wild type grown under same conditions (*P* < 0.01, Welch’s *t* test).

To increase the heterocyst frequency of Anabaena, we first knocked out the two genes encoding suppressors of heterocyst differentiation, HetN and PatS. Both proteins are known to inhibit heterocyst differentiation in an independent manner; PatS contributes at the initial stage of heterocyst development and HetN maintains the ratio between heterocyst and vegetative cells under nitrogen-starved conditions. As shown in Figures 1A,B, the double null mutants of *hetN* and *patS* (Δ*hetN*/*patS*) were isolated. A PCR analysis using primers external to *hetN* (Figure 1A, #1 and #4) resulted in two PCR fragments of different sizes. The wild type strain produced a 2,238 bp fragment, and the mutant produced a larger 3,641 bp fragment because of the presence of the spectinomycin resistance cassette. PCR with a primer that annealed to the middle region of *hetN*, #14, produced an amplicon only from the wild type genome as the necessary region was not present in the mutant genome. PCR with primer #13, which anneals to the spectinomycin resistance cassette, produced amplicons only from the mutant genome. These results indicated that no wild type copies of *hetN* were present in the mutant cells. Similar analysis was conducted for *patS*, and null mutation was also confirmed. There were no obvious differences of growth between the double mutant and wild type under nitrogen-replete conditions. Under nitrogen-starved conditions, however, the double mutants exhibited retarded growth, especially from the second day after nitrogen step-down (Figure 1C). This is probably because the double mutant required extra energy to develop more heterocyst cells compare to the wild type. Lipid analysis showed that the double mutants accumulate more Hgl compared with the wild type (Figure 2A). A thick band of Hgl was seen in the double mutant after 48 h of nitrogen step-down, whereas a faint band in the wild type appeared under same conditions. We also analyzed heterocyst frequency of the double mutants. Compared with the wild type, similar differentiation rate was observed in the double mutants after 24 h, but they showed approximately 3.5-fold more heterocyst cells after 48 h of nitrogen step-down (27.2%, Figure 2B). These results imply that knockout mutations of *hetN* and *patS* can enhance the accumulation of Hgls and/or their precursor, fatty alcohols. It is of note that our Δ*hetN*/*patS* double mutant could grow diazotrophically, which is not same as the previously reported Δ*hetN*/*patS* mutants (Corrales-Guerrero et al., 2014).

### Triple Mutant of *hetN, patS*, and *hglT* Showed Lower Growth Rate, Chlorophyll Content, and Photosynthetic Activity Under Nitrogen-Starved Conditions

**FIGURE 3:**
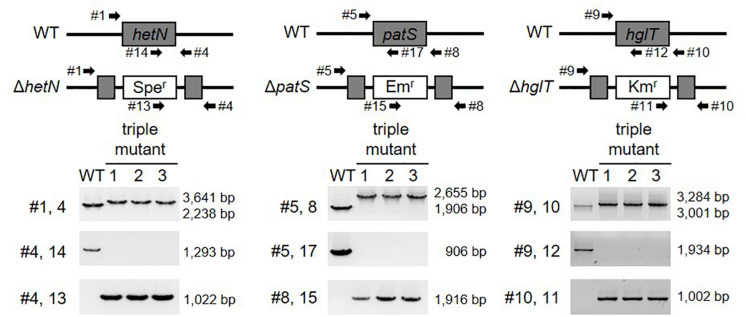
Genotype of *hetN*/*patS*/*hglT* triple mutants. Upper layer shows schematic representations of *hetN*, *patS*, and *hglT* regions of the wild type and mutant genomes. Bottom layer shows genotype analysis of the triple mutants using the primers highlighted by arrows in the upper layer. Spe^r^: spectinomycin resistance cassette, Em^r^: erythromycin resistance cassette, Km^r^: kanamycin resistance cassette.

**FIGURE 4:**
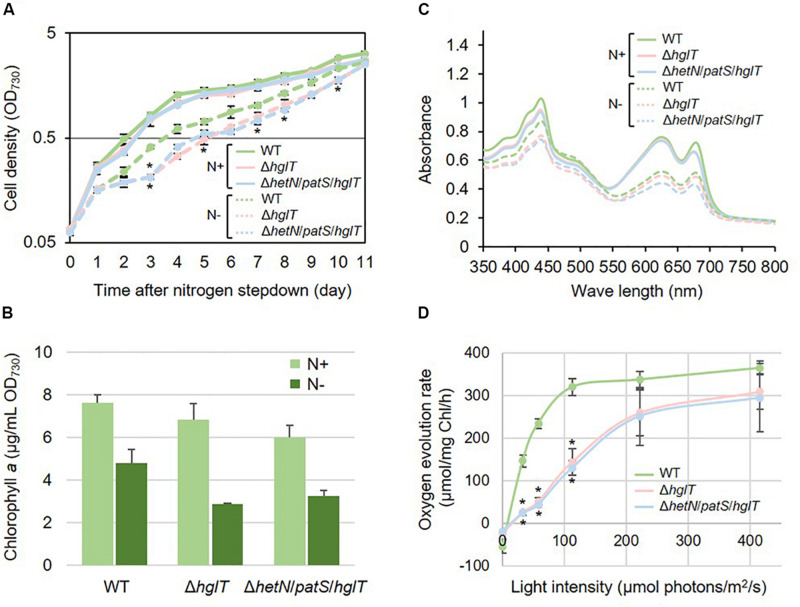
Growth curve, chlorophyll contents, and cell spectra of *hglT* mutants. The wild type and mutant cells were grown in BG11 (N+) or BG11_0_ [N–, 48 h of nitrogen step-down in **B,C**]. **(A)** Growth curve of the *hglT* mutants. Solid line: N+, hashed line: N–. Error bars indicate the SD based on three independent experiments. Asterisks indicate significant differences from the wild type grown under same conditions (*P* < 0.01, Welch’s *t* test). **(B)** Chlorophyll *a* contents of the wild type (WT), *hglT* single mutant (Δ*hglT*), and *hetN*/*patS*/*hglT* triple mutant (Δ*hetN*/*patS*/*hglT*). Error bars indicate the SD based on three independent experiments. **(C)** Cell spectra. Solid line: N+, hashed line: N–. Representative data are shown. **(D)** Oxygen evolution rate. Error bars indicate the SD based on three independent experiments. Asterisks indicate significant differences from the wild type at the same light intensities (*P* < 0.01, Welch’s *t* test).

Next, we tried to knockout *hglT* in Δ*hetN*/*patS*. Figure 3 shows the results of genotyping of the isolated triple mutant of *hetN*, *patS*, and *hglT* (Δ*hetN*/*patS*/*hglT*). Similar genotyping analyses as described above were conducted for *hetN*, *patS*, and *hglT*, and null mutation of each gene was confirmed. Using these triple mutants, first the growth rates were compared with the wild type and single *hglT* mutants. There were no obvious differences among these three strains under nitrogen-replete conditions. Under nitrogen-starved conditions, however, both the single and triple mutants exhibited retarded growth, especially from the second day after nitrogen step-down (Figure 4A). There are probably two parallel reasons to explain this growth retardation in the triple mutant. First is that, as was seen in the Δ*hetN*/*patS* double mutants, more energy and carbon sources were used for development of heterocyst cells, rather than cell divisions. Second is that the accumulated aglycones (fatty alcohols) of Hgls by the *hglT* mutation are not sufficient to complement functions of Hgls and lower the efficiency of nitrogen fixation in the heterocyst cells (Halimatul et al., 2014). Δ*hetN*/*patS*/*hglT* had less chlorophyll *a* compared with the wild type under both nitrogen-replete and -starved conditions (Figure 4B), which can be also seen in the cell spectra (Figure 4C: 678 nm peak). The cell spectra of both Δ*hglT* and Δ*hetN*/*patS*/*hglT* mutant cells showed no significant alteration in the phycocyanin peak (625 nm) under nitrogen-replete conditions compared with the wild type. Under nitrogen-starved conditions, 625 nm peaks were reduced in both single and triple mutants, which fit to the observation of delayed growth in these mutants (Figure 4A). Because the chlorophyll content was low in the mutants, photosynthetic activity was measured (Figure 4D). Both the Δ*hglT* and Δ*hetN*/*patS*/*hglT* cells showed retarded photosynthetic activity, especially under low light conditions, such as ∼100 μmol photons/mg Chl/h. The values are around five times lower in the mutants at low light conditions (30–60 μmol photons/mg Chl/h) but around 1.2 times lower at middle to high light conditions (200–400 μmol photons/mg Chl/h). Oxygen evolution rates of the single and triple mutant cells were very similar, indicating that their retarded photosynthetic activity is due to the inactivation of *hgl*T, not *patS* and/or *hetN*.

### *hetN/patS/hglT* Triple Mutants Exhibited More Heterocysts but Little Accumulation of Fatty Alcohols

**FIGURE 5:**
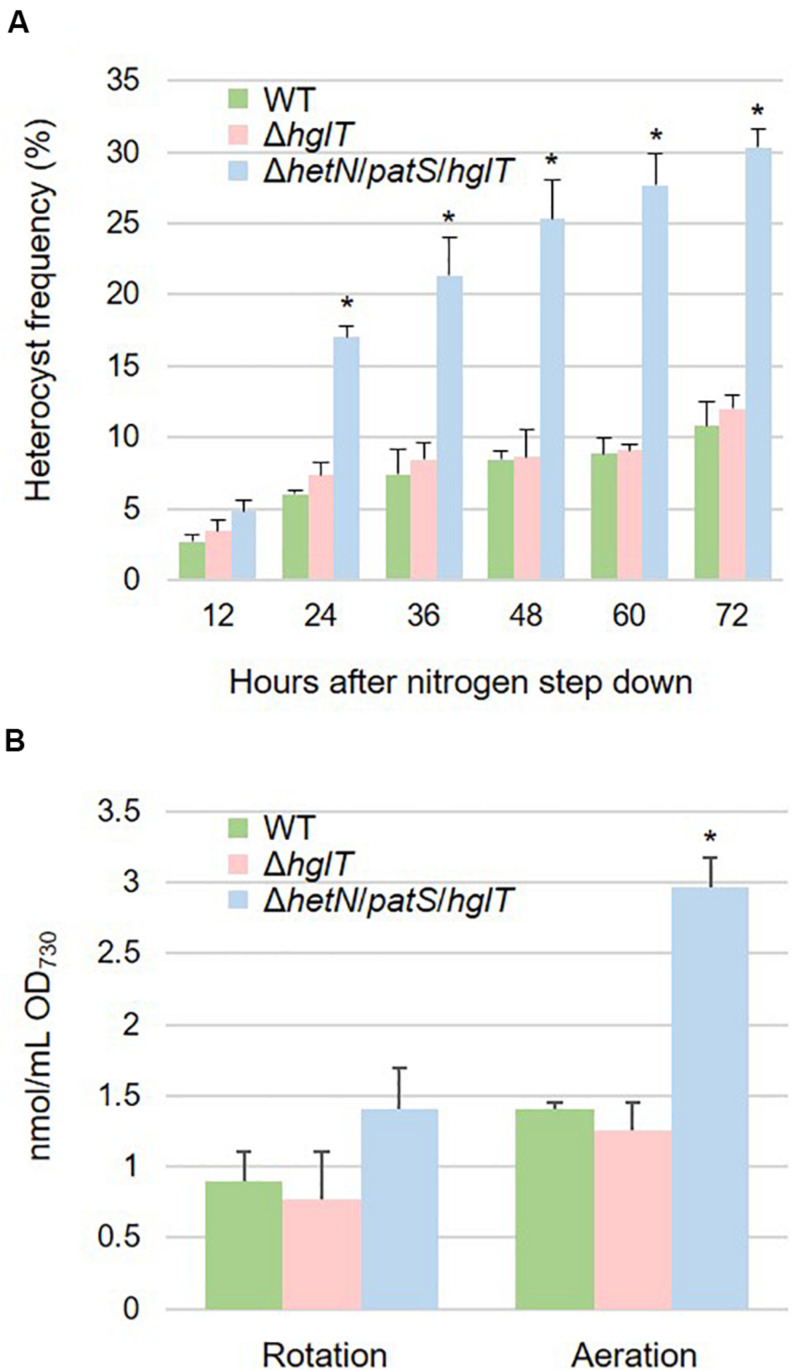
**(A)** Heterocyst frequency of the wild type (WT), *hglT* single mutant (Δ*hglT*) and *hetN*/*patS*/*hglT* triple mutant (Δ*hetN*/*patS*/*hglT*). Error bars indicate the SD based on three independent experiments. Approximately 500 cells were counted per experiment. Asterisks indicate significant differences from the wild type at the same timepoints (*P* < 0.01, Welch’s *t* test). **(B)** Accumulation of the heterocystous lipids. Wild type accumulated HGLs while the mutants accumulated aglycones (fatty alcohols). Error bars indicate the SD based on three independent experiments. Rotation: cells cultured on rotary shaker in Erlenmeyer flasks, Aeration: cells cultured in test tubes with bubbling. An asterisk indicates a significant difference from the wild type grown under same conditions (*P* < 0.01, Welch’s *t* test).

Heterocyst frequency of the Δ*hetN*/*patS*/*hglT* triple mutant was analyzed. Figure 5A shows that the triple mutant exhibited a higher heterocyst differentiation rate (approximately three times) compared with the wild type and Δ*hglT* single mutant, especially after 24 h of nitrogen step-down. The heterocyst frequency kept increasing; after 72 h, the rate stabilized at 30.3%, which was much more than the wild type (10.8%) and Δ*hglT* single mutant (12.0%). Since the triple mutant had a high heterocyst frequency, we analyzed the expression level of *nifH*, the gene encoding a subunit of nitrogenase that is specifically expressed in heterocyst cells. As shown in Supplementary Figure S1, mRNA level of *nifH* was similar among the wild type, Δ*hglT* mutant, and Δ*hetN*/*patS*/*hglT*. This result indicates that the triple mutant cells have less nitrogenase per heterocyst cell, at least the expression of *nifH*, because they have three times higher rate of heterocyst formation compared with the wild type and Δ*hglT* single mutant; the expression rate of *nifH* remained the same. We then finally analyzed lipid composition of the mutants. As seen in Figure 5B, the triple mutant harbored 1.41 nmol/mL OD_730_ of fatty alcohols. This was about 157% of the accumulation of Hgls in the wild type (0.90 nmol/mL OD_730_). Since the heterocyst frequency was approximately three times higher than the wild type, it was about half less the amount we expected.

### Aeration of Culture Enhanced Accumulation of Fatty Alcohol in the *hetN/patS/hglT* Triple Mutant

To enhance the accumulation of fatty alcohols in Δ*hetN*/*patS*/*hglT*, we optimized the cultural conditions. Since the experiments described above were conducted using a rotary shaker, we speculated whether the efficiency of Hgl and its aglycones, fatty alcohols, can be boosted by aeration, since aeration in general has been shown to promote better cyanobacterial growth than rotation. As expected, the cells cultured with aeration showed higher accumulation of Hgls and aglycones than those cultured by rotation (Figure 5B). Accumulation of Hgl in the wild type increased to 1.5-fold compared with the cells cultured by rotation (1.41 nmol/mL OD_730_). This increase was also seen in the *hglT* single mutant (0.77–1.25 nmol/mL OD_730_). In the triple mutant, the amount of fatty alcohols increased to 2.97 nmol/mL OD_730_, which is more than twice that of the cells cultured by rotation.

## Discussion

### Aeration Might Boost the CO_2_ Uptake and/or Oxygen Diffusion Into the Cells

In this study, we initially used rotary shakers to grow cells of cyanobacteria. However, we found that the effectiveness of lipid production is dependent on the degree of aeration. The mutants cultured with continuous aeration accumulated fatty alcohol with a maximum production rate, over twice that obtained by rotary shaking (Figure 5B). This might be because influx of CO_2_ from aeration increased the intracellular C/N ratio and induced the accumulation of carbon products in the cells. Another possibility is that aeration led to oxygen saturation in the culture, which led the thickening of the heterocyst envelope, especially the glycolipid layer. It has been reported that when the heterocyst containing cyanobacteria (*Anabaena flos-aquae*) are cultured in high partial pressure of oxygen under nitrogen-starved conditions, their heterocyst cells develop an envelope with a very thick HGL (Kangatharalingam et al., 1992). Therefore, it is possible that the Δ*hetN*/*patS*/*hglT* cells are able to sense oxygen concentration in the culture and develop a thicker envelope under aeration. We have not tried the additional CO_2_ and/or O_2_ in the air for bubbling yet, but it would be interesting topic for future investigation.

### Possible Modifications to Enhance Fatty Alcohol Production

Culturing the *hetN*/*patS*/*hglT* triple mutant cells with bubbling led to an accumulation of 2.97 nmol/mL OD_730_ fatty alcohols. This amount is equivalent to 1.23 mg/L OD_730_ and lower than those previously reported for the production of fatty acids in other cyanobacteria. For example, genetically modified *Synechocystis* PCC 6803 has been reported to synthesize >200 mg/L of fatty acids (Eungrasamee et al., 2019). Kato et al. (2017) reported that the removal of the product from the medium enhances bioproduction in cyanobacteria. They overlaid isopropyl myristate to remove the toxic product, palmitic acid, from the medium of the unicellular cyanobacterium *Synechococcus elongatus* PCC 7942 and found that this two-phase cultural system also accelerated the production of the fatty acid. To adopt this two-phase system, it was very important to let our cells secrete the product into the growth medium. It has been proposed that Hgl aglycones localize to the space between the outer membrane and the heterocyst envelop polysaccharide (HEP) layer and as do the Hgls in the wild type (Halimatul et al., 2014). The disruption of HEP layer in our mutants may allow the cells to secrete the fatty alcohol into the medium under nitrogen-starved conditions and enable its extraction into the overlaid solvent, such as isopropyl myristate.

### Anabaena Is a Useful Platform for Bioproduction

Anabaena can fix gaseous nitrogen, an ability that makes them a great candidate for use as a platform for bioproduction. For example, ethanol production using heterocyst cells has been reported by Ehira et al. (2018). They reported more than a hundred times yield (∼170 mg/L) compared with anthropogenic production, indicating that Anabaena has a huge potential for bioproduction. Fatty alcohol in this report was synthesized through reactions of polyketide synthases (PKS). Presently, PKS and non-ribosomal peptide synthase (NRPS) are in the spotlight. In cyanobacteria, PKS has been known to synthesize toxic compounds such as microcystin (Shishido et al., 2019) and swinholide (Humisto et al., 2018). In some marine bacteria, PKS synthesize very long chain fatty acids, such as docosahexaenoic acids (Okuyama et al., 2007). Recently, NRPSs have successfully been expressed in Anabaena for bioproduction (Videau et al., 2016, 2019). The replacement of genes involved in the synthesis of Hgl with desired PKS/NRPS will be of great interest.

## Data Availability Statement

All datasets generated for this study are included in the article/Supplementary Material.

## Author Contributions

HM and KA conceived the research and wrote the manuscript. HM and EA conducted the experiments. HM, EA, and KA performed the data analysis. All authors contributed to the article and approved the submitted version.

## Conflict of Interest

The authors declare that the research was conducted in the absence of any commercial or financial relationships that could be construed as a potential conflict of interest.

## Supplementary Material

FIGURE S1 Transcript level of *nifH* under nitrogen starvation. The relative quantities of *nifH* were determined by qRT-PCR in the wild type (WT), *hglT* single mutant (Δ*hglT*), and the triple mutant for the indicated time. Values are expressed as the means of duplicated experiments.
