# Supplementary figures and images for: hetN and patS Mutations Enhance Accumulation of Fatty Alcohols in the hglT Mutants of Anabaena sp. PCC 7120
Source: Front Plant Sci. 2020 Jul 8;11:804. doi: 10.3389/fpls.2020.00804 (PMC7360850; doi:10.3389/fpls.2020.00804)

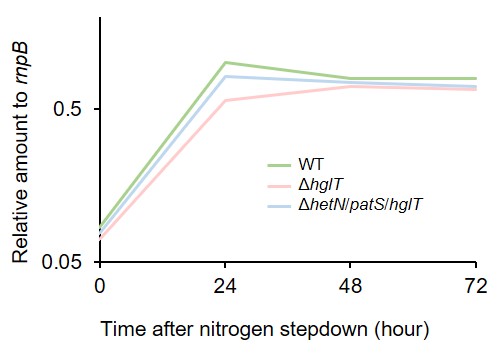

Supplement: FIGURE S1 — Transcript level of nifH under nitrogen starvation. The relative quantities of nifH were determined by qRT-PCR in the wild type (WT), hglT single mutant (ΔhglT), and the triple mutant for the indicated time. Values are expressed as the means of duplicated experiments. [file Image_1.JPEG]
