# Supplementary material for: hetN and patS Mutations Enhance Accumulation of Fatty Alcohols in the hglT Mutants of Anabaena sp. PCC 7120
Source: Front Plant Sci. 2020 Jul 8;11:804. doi: 10.3389/fpls.2020.00804 (PMC7360850; doi:10.3389/fpls.2020.00804)
Supplement: Supplementary file 2 [file Table_1.DOCX]

Supplementary Table 1. Primers used in this study.

| # | Primer name | Sequence |
| --- | --- | --- |
| 1 | HetN KO 5’ Fw | ACTAGTGGATCCCCCGATTTGTACCTCAAAG |
| 2 | HetN KO 5’ Rv | GAATTCCTGCAGCCCAAAGCACGAGCAATGTAG |
| 3 | HetN KO 3’ Fw | TCGACCTCGAGGGGGACAAATTTCTCCCACTTC |
| 4 | HetN KO 3’ Rv | GCGAATTGGGTACCGCTACCTTATCTAGTGAC |
| 5 | PatS KO 5’ Fw | GCGAATTGGGTACCGCCTCGTGAAGCATG |
| 6 | PatS KO 5’ Rv | TCGACCTCGAGGGGGTGATATCTAGGAAGTTGG |
| 7 | PatS KO 3’ Fw | GAATTCCTGCAGCCCTGCCTCGACTATCGG |
| 8 | PatS KO 3’ Rv | ACTAGTGGATCCCCCGACTCTGGGAGTAAATTG |
| 9 | HglT KO 5'Fw | ACTAGTGGATCCCCCTCTGACAAATCCGACG |
| 10 | HglT KO 3'Rv | CGGGCCCCCCCTCGAAGTTTTAGCCACAGTTC |
| 11 | Km up | CCGCTTCCTTTAGCAGC |
| 12 | hglT Rv | ACTACTGGAGTACCAGAG |
| 13 | spec-mid-Rv | ATCACGGCACGATCATCGTG |
| 14 | hetN Fw | CCAGCAGGCTCAAGATATTG |
| 15 | Em-Fw | AAGCTTCGCGTGCTATAATTATACTAA |
| 16 | PatS-Fw3 | ACTACCGCGCTCATCACAG |
| 17 | PatS-Fw | CCGCGCTCATCACAGAAAT |
| 18 | nifH_Fw | ACCTCGTGACAACATCGTTC |
| 19 | nifH_Rv | TTGGTGTAGGAATGGTGAGC |
| 20 | rnpB-RTFw | CCAGTTCCGCTATCAGAGAG |
| 21 | rnpB-RTRv | GAGGAGAGAGTTGGTGGTAAG |
